# Demand-side challenges to increase sales of new maize hybrids in Kenya

**DOI:** 10.1016/j.techsoc.2021.101630

**Published:** 2021-08

**Authors:** Pieter Rutsaert, Jason Donovan, Simon Kimenju

**Affiliations:** a International Maize and Wheat Improvement Centre (CIMMYT), Nairobi, Kenya; b International Maize and Wheat Improvement Centre (CIMMYT), Texcoco, Mexico; c Kula Vyema Centre of Food Economics, Nairobi, Kenya

**Keywords:** Seed systems, Consumer behavior, Agro-dealers, Varietal turnover, Demand orientation

## Abstract

In Eastern and Southern Africa, as the public sector has retreated from maize seed production and the private sector has emerged to fill the resulting void, a key issue for researchers, governments and private donors has been the capacity of privately owned, typically small scale, seed businesses to effectively produce and distribute hybrid seed. Roughly two decades on, research and development programming continues to focus on supply side issues in supporting the maize seed industry to bring new varieties to farmers. Motivated by thinking on agri-food value chains, this article explores the potential for achieving a stronger demand orientation in programming to support the maize seed industry, a requirement for varietal turnover. In 2019 data were collected in Kenya from i) 80 agro-dealers on their relations with seed businesses and their marketing of maize seed, ii) 466 farmers on their seed choice and engagement with agro-dealers and iii) 8 seed companies on their distribution and sales strategies. Results confirmed the overarching supply-push orientation of the industry, characterized by limited innovation and risk taking, weak collaboration between actors, low margins for retailers, and limited investments in seed marketing. Farmers showed weak appetite for acquiring new seed products, preferring instead to purchase seeds that they knew from experience. Better strategies for building seed value chains will require deeper insights on stakeholders needs and strategies, to include the capacity of seed businesses and retailers to innovate in business management and marketing.

## Highlights

• Strategies and actions to accelerate varietal turnover will benefit from a stronger demand orientation.
• Seed companies face weak incentives and limited capacities to market new seed.
• Agro-dealers, with weak links to seed companies, do not actively introduce new varieties to farmers.
• Farmers are reluctant to seek out new varieties, preferring to remain with the varieties they know and trust.
• A value chain approach offers potential to address these issues, but critical information gaps remain.

## 1 Introduction

Smallholder adoption of modern production inputs comprises a key component of strategies for increased agricultural productivity, and thus an important contribution to the larger development goals related to poverty reduction, food security and nutrition. Following the Asian Green Revolution, tremendous effort has been made in promoting the adoption of improved seeds and fertilizer by smallholders in Sub-Saharan Africa [[1], [2], [3]]. Given the outsized role that maize plays in human diets in Eastern and Southern Africa, governments and donors have sought to expand the availability of new maize hybrids for smallholders [4,5]. Besides breeding, attention has been given to seed delivery, including the capacity of emerging seed businesses to produce quality seed for the retail market. Over the past two decades, governments have grown increasingly reliant on the private sector for maize seed multiplication and delivery, although public sector engagement remains important in breeding and quality certification. Important among private sector stakeholders are seed companies that multiply and distribute seed and agro-dealers of various shapes and sizes that sell seed and other inputs to farmers.

**Fig. 1:**
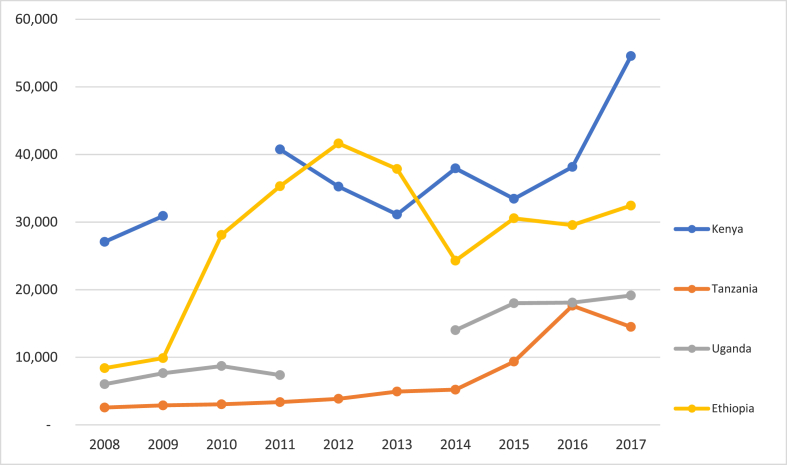
Certified maize seeds production volumes from 2008 to 2017.

Source: Kenya, KEPHIS Annual Reports; Tanzania, TOSCI; Ethiopia, Ministry of Agriculture and Natural Resource, Plant Variety Release, Protection and Quality Control Directorate's, Tasai, Uganda brief 2018; Uganda Early Generation Seed Study (2016); Baseline Survey of the Seed Sector in Uganda (2018)

Roughly 20 years ago, the transition in the region from a public-sector to a private-sector driven maize seed sector necessitated a prioritization of investments in building reliable sources of quality seed [6,7]. Given the crucial role played by privately owned seed companies in the process, assessing their growth and development, including their capacity to deliver increasingly higher volumes of hybrid maize seed, justifiably commanded the attention of researchers interested in formal maize seed systems [[8], [9], [10], [11], [12]]. Overall, it can be argued that donor and private sector investments in building the market for hybrid maize seed in Eastern and Southern Africa have been successful. In most of the countries there are dozens of seed companies in operation [9], that are supported by seed sector associations [13] and national agricultural research centers that deliver intensive training and education programs for seed production [14]. As the maize seed industry has grown, so too have the volumes of seed made available to farmers, with volumes doubling or even tripling in East Africa over the last decade (Fig. 1). Work by Abate et al. [15] estimated a coverage of roughly 82% of Eastern Africa and 55% of Southern Africa with improved maize cultivars in surveyed areas.

However, from the perspective of donors, government agencies and some seed companies, the sector has struggled increasingly with what can be considered a second-generation challenge for the development of formal maize seed systems: slow varietal turnover, or the tendency of seed companies to prioritize resources for the production and distribution of well-known, older hybrids over lesser-known, newer hybrids. The varietal turnover issue is especially acute when considered in light of the strong investments in Sub-Saharan Africa to advance the production of maize varieties that are more resilient to the effects of climate change [16,17]. Scores of new hybrids have been advanced by the CGIAR and national breeding programs that perform under drought, low nitrogen as well as disease stress such as Maize Lethal Necrosis (MLN) disease and striga [[18], [19], [20]]. On-farm trials have suggested that these hybrids deliver higher yields than popular hybrids that are currently on the market both under stress as well as normal conditions [21,22] and several studies have confirmed the positive impact of improved varieties on household food security [[23], [24], [25]]. However, it has also been noted that outdated maize hybrids continue to dominate the market across many African countries [26]. Moreover, the seed industry has shown muted enthusiasm for the introduction of maize hybrids with unique nutrition attributes, such as Quality Protein Maize or provitamin A-rich orange maize [[27], [28], [29]]. This is despite various studies that have demonstrated consumer interest in these products [[30], [31], [32]].

Looking ahead, a key issue facing private donors, governments, and others interested in the diffusion of technologies to smallholders by the private sector is the capacity, incentives, and barriers for the businesses to effectively introduce new products into the market. How best to do this across different contexts remains an open question. Overall, Eastern and Southern African seed producers and retailers operate in a complex business environment, which translates into increased uncertainty along the development pathway (e.g. lack of access to finance and other services, and a long history of seed subsidy programs). Moreover, competition within the seed industry has been growing, spurred by the growing presence of multinational companies and the increasing capacity of locally owned companies. While farmers have a wide range of seed products to choose from, they have shown limited interest in experimenting with new seed products [33,34]. Insights on farmer decision making on the other hand remain scarce, as does reliable and timely market intelligence. Despite these demand-side challenges, support for maize seed systems development continues to focus attention on the production of seed by locally owned seed companies.

This paper looks to shed new light on possible entry points for future interventions in support of formal maize seed systems development in Kenya. In recognition of the second-generation challenges around competitiveness, marketing, and consumer demand, we adapt a value chain perspective which focuses attention on the strategies, incentives and needs of actors in the chain and the relationships between them. The rest of this paper is organized as follows. The next section presents the conceptual framework and discusses supply and demand orientation in the maize seed industry. Section three outlines the data collection methods, including an agro-dealer survey, farmer intercept interviews and seed company interviews carried out in Kenya. Section four reports the findings on different elements of value chain dynamics including (i) seed company engagement in retail and distribution, (ii) supplier influence on agro-dealers, (iii) agro-dealer challenges and priorities to increase sales, (iv) store environment and (v) the relationship between agro-dealers and farmers. In the final section we summarize the bottlenecks for seed value chain development and discuss ways forward to increase demand driven thinking.

## 2 Conceptual framework

### 2.1 Supply orientation in maize seed systems development

Morris et al. [35] first conceptualized the development pathway of the maize seed industry in developing countries. They considered a linear growth trajectory, whereby the industry passes through preindustrial, emergence and expansion phases before it reaches the mature phase. Over time, seed businesses were projected to grow in size and capacity, hybrids were to overtake open pollinated varieties, and the government role in the seed sector was to diminish. With each advance in phase, seed producers were expected to show higher levels of capacity and professionalization. The same trend was expected in farming practices in the form of increased commercial-oriented farming and higher rates of adoption of hybrid seed, to include annual seed repurchasing. While over 20 years old, the framework continues to influence thinking on how the maize seed industry develops, as evidenced by recent reports of Alliance for a Green Revolution in Africa (AGRA) and the African Development Bank (ADB) [36,37]. Where advances in seed systems development hinges on making available greater volumes of hybrid maize seed, such as in parts of Western Africa (e.g. Refs. [38,39], this framework continues to provide a foundation for conceptualizing intervention entry points.

However, across large parts of Eastern and Southern Africa, Latin America and Southern Asia, evidence suggests that locally owned seed industries have acquired the capacity to produce and deliver hybrid maize seeds. This is attributable, in part, to decades of previous government and private donor investments in building their supply capacity [5,40,41]. This suggests the need for new thinking on how to address demand side challenges in maize seed systems. For example, the expansion of seed products launched onto seed markets where hybrid adoption is already widespread increases competition between locally owned seed companies, many of which are still attempting to consolidate their business and expand their seed sales. Moreover, strong demand for improved varieties has the potential to encourage the expansion of multinational seed companies, as shown by the recent acquisitions in Eastern and Southern Africa, to include Syngenta's acquisition of MRI Seed Zambia Ltd in 2013 and Pioneer's (now Corteva) merger with Pannar seeds in 2012. In some cases, governments have strategically invested in locally owned seed businesses with the expectation that they will reduce dependence on imported seed and seed produced by multinational companies [42]. Work on value chains has already highlighted these complexities, including non-linearity in development pathways [43].

Another factor that has yet to be addressed in frameworks on formal maize seed systems development are the challenges for the private sector to absorb (and benefit from) constant technological advancements in breeding. With the continuous focus on genetic gains and higher stress resistance in product innovation, seed companies are confronted with the tradeoff of investments in innovation and the accompanying financial risks and marketing challenges versus maintenance (or expansion) of the market share of hybrids already on the market. Discussions on how small and medium enterprises (SMEs) overcome the managerial, marketing and technical barriers to launching new products has featured prominently in the business management literature (e.g. Ref. [44]. In the broader literature on small and medium enterprise (SME) development, economists have pointed out the challenges for SMEs to innovate under competitive marketing conditions [45]. New frameworks are required to better understand the unique set of opportunities and barriers faced by seed companies to produce new seeds and address the ‘consumers’ burden ‘(i.e. the time, effort and purchase risk faced by farmers in the adoption of new seeds).

Discussions have recognized the need for action to support increased adoption of new hybrid maize seeds; however, the solutions proposed tend to focus exclusively on supply-related strategies and are based on strong assumptions about how markets function. For example, Smale et al. [46] discussed the challenges faced in the introduction of provitamin A-rich orange maize in Zambia but their work focused mainly on varietal release strategies (e.g. exclusive production and distribution rights to select seed companies). Others have pointed to the possibility that promoting strong competition among seed companies would foster investment in the production and marketing of new varieties, based on experiences in the United States where maize hybrids tend to be phased out and replaced by the private sector every three to four years [16,47]. Researchers have also suggested the need to better leverage agricultural extension services, based on the notion that insufficient information about new hybrids was a major bottleneck for greater adoption by smallholders [48,49].

However, whether small and medium-sized maize seed businesses in Eastern Africa are able to deliver innovation in seed genetics in shorter time frames to smallholders remains an open question. Among the challenges they face are higher costs (vis-vis larger seed businesses), cash-strapped retailers operating largely in the informal sector, and farmers who are ill-informed about the seed options available. Others have suggested that innovation in policies will drive faster varietal turnover, to include the obligatory retirement of older maize varieties or accelerating the registration and release processes for new cultivars [50,51]. Notwithstanding the need for strong and adaptive seed companies as well as the right policy environment, we argue that at this juncture a more drastic shift in seed systems thinking will be necessary with farmer demand as the entry point for the formulation of strategies to support seed industry development.

### 2.2 ‘Supply chains’ versus ‘value chains’ and relevance for maize seed industry

Beginning in the early 2000s, development economists began writing about the structural changes in global agri-food markets and the implications for producers, processors, and retailers (for review, see Ref. [52]. In exploring the potential marketing opportunities for smallholders in tropical countries, development economists distinguished between ‘supply chains’ (networks of businesses oriented towards delivering high volume at low cost) and ‘value chains’ (networks oriented towards meeting consumer demand for higher quality and other attributes). Where supply chain thinking tended to focus on efficiency in production and distribution, reducing costs and protection of data and information, value chain thinking focused on adding value, market segmentation and product differentiation (e.g. quality, certification), collaboration with shared risks and benefits and end-user focus [53,54]. Instead of looking at a single actor (e.g. seed companies in the case of seed industry development), the value chain framework considered the range of stakeholders and their successive value-adding activities of getting the product from conception to the user should be seen as one system with the focus on the end-user [55]. Value chain concepts have been applied extensively over the past two decades for the design of development programming that seeks to link smallholders to more lucrative markets in pursuit of poverty reduction and other goals [[56], [57], [58]].

**Fig. 2:**
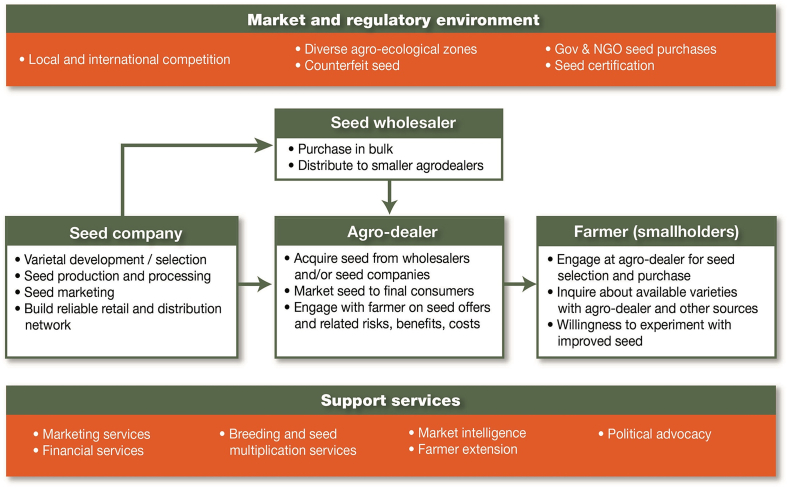
Stylized maize seed value chain linking seed companies to smallholders.

Bringing a value chain approach to the discussions on marketing by the maize seed industry (and related discussions on accelerating varietal turnover) will require a much better understanding on two fronts: strategies, capacities, and needs of seed businesses and retailers as well as a better understanding of farmer needs. Fig. 2 portrays a stylized maize seed value chain, linking seed companies to smallholders, complemented with the marketing and regulatory environment as well as the support services needed.

To minimize transaction costs, seed companies sell directly to a small number of wholesalers or large agro-dealers, that in turn, interact with smaller village-based agro-dealers that sell the seed to farmers. However, very little is known about the challenges currently present in those distribution networks. Rutsaert and Donovan [59] found that seed companies depended on agro-dealers to sell their products but focused their marketing activities directly towards farmers through radio and demonstration plots, altogether bypassing retail networks. Their overall strategy relied on simulating farmer demand before the farmer arrives at the retailer to purchase maize seed. Research interest in agro-dealers has mainly revolved around their role in input subsidy programs [2,60]; however, evidence on agro-dealer functioning and decision-making remains scarce. Agro-dealers are often small businesses [61], dealing with high competition, low profit margins and chronic cash constraints [62]. Under these survival circumstances, management decisions are likely to be intuitive, short term and less strategic [45]. However, there is currently no available evidence on their views on hybrid maize seed, interest in increasing varietal turnover or capacity to become a reliable seed value chain partner.

Value chain development is more likely when the overall regulatory and marketing environment are conducive to business growth and investment. However, distortions in seed markets resulting from onerous and counterproductive seed regulations increase the burden on seed producers [5,51]. Sub-Saharan Africa has a long history of subsidy or relief programs for agricultural inputs like seed and fertilizer [2,5,63]. As described by Tripp and Rohrbach [5]; agricultural input support in the form of free or subsidized seed are often initiated through relief programs initiated by disaster, such as drought. But after the initial relief program, subsidized input provision tends to continue, either as part of poverty alleviation strategy or because of political motivations. While these programs can provide short-term benefit to farmers (low-cost or free seed) as well as seed producers (bulk sales to NGOs, governments or relief programs), they also undermine the long-term viability and development of maize seed value chains. Not only do retail networks face unfair competition leading to crowding out [46], the diversion of maize seed to relief programs also endangers steady supplies for commercial networks. Another well-known challenge for value chain development is the prevalence of counterfeit seed in the market [47,64,65]. Although a recent study by Barriga and Fiala [66] in Uganda testing seed quality along the supply chain counters this argument and highlights that low quality seed appears to be caused by mishandling and poor storage of seed.

Crucial for the growth and development of the African maize seed industry is the establishment of support services, including financial services, business advisory services, and production-related services. One example in recent years has been private donor investments in establishing providers of early generation maize seed [67,68]. Access to high quality parent material such as foundation seed, which seed companies need to start their seed production, has been identified as a primary bottleneck for seed multiplication in Africa [69], resulting in targeted private donor support as neither the public nor the private sector were equipped to invest in this. Other efforts to increase seed production focused on simplifying regional trade through harmonized seed laws [70], capacity development in breeding and crop production [14] and setting up seed companies [71]. Further downstream, investments have focused on better functioning distribution networks through agro-dealer development including capacity building, facilitating access to credit, strengthening agro-dealer associations, bringing agro-dealers closer to farmers and demand creation activities, i.e. supporting demonstration plots for hybrid seed promotion [72]. All these investments have focused on the establishment of a functioning seed supply chain that is capable of bringing affordable quantities of improved seed to farmers. However, faster innovation processes will require reliable access to different types of support services over time, including those related to marketing, finance, and business management.

Effective value chain development strategies will rely, in part, on stakeholders understanding the needs and interests of key actors along the chain, to include final consumers. One of the key pillars in the theory of change around varietal turnover has been that farmers will replace cultivars if they are confronted with the superiority of new planting material. However, this assumption has been contested by different researchers that question if farmers actually base their seed choice on a rational assessment of traits that maximize their utility. Fitzgerald [73], for example, argued that the transition from open-pollinated varieties, where farmers selected the best strains suited to their own farming conditions, to hybrids increased the dependence on the advice of seed suppliers. Stone [74] noticed that farmers attending demonstration plots in India could recall other farmers who grew or promoted the seed, but rarely recalled agronomic details of the actual plot. Kilwinger et al. [75] found that banana farmers in Uganda associated seed attributes with the source where they acquired that seed. Rutsaert and Donovan [59] highlighted that when agro-dealers engaged with farmers who were buying seed, they had a strong influence on the purchase decision. These findings stress the need for a better understanding of how farmers make their seed choices and the up-stream engagement they have with retailers.

Given the overall lack of knowledge about the demand side of the maize seed industry in Sub-Saharan Africa, there are limited insights upon which to begin a meaningful discussion on possible entry points for supporting maize seed value chain development. This paper aims to contribute to this knowledge gap by answering the following questions: i) what are the incentives, capacities and limitations of seed companies to build their business and expand their market, ii) what challenges do retailers face to support innovation and value adding in the hybrid seed market, and iii) how do farmers decide on seed purchases at the agro-dealer and what influences their varietal selection.

## 3 Materials and methods

### 3.1 Maize seed production and distribution in Kenya

Following the liberalization of the Kenyan seed sector in the 1990s, dozens of domestically-owned private seed companies emerged together with international companies [41,76]. According to The African Seed Access Index [77], 19 companies produced and distributed maize seed in Kenya comprising ten private domestic companies, eight international companies and the parastatal Kenya Seed Company in 2015. With the large number of players, the Kenyan maize seed market is considered to be the most crowded in the region, with a diversity in marketed varieties far exceeding that in surrounding countries. Farmer adoption data from 2009 reported 60 different hybrids being grown by Kenyan farmers, compared to 27 in Tanzania, 16 in Ethiopia and 11 in Uganda [78]. These numbers coincide with a recent adoption study that focused on 10 maize-growing counties in Eastern Africa, reporting 41 hybrids in Kenya against 17 in Tanzania, 12 in Ethiopia and nine in Uganda [15].

Despite experimentation with various other distribution models including public sector distribution and supplying seed through village-based advisors, the agro-dealer model remains the most prevalent form of seed distribution in Kenya [61, 8]. Given the importance of retailers in the distribution of maize seed and other inputs, several development programs have focused their efforts on agro-dealer development. Cultivating New Frontiers in Agriculture (CNFA) and the Alliance for a Green Revolution in Africa (AGRA) have supported the professionalization of agro-dealers [72,79]. Through the Kenya Agro-dealer Strengthening Program (KASP) and other programs (i) over 3000 agro-dealers were trained in business management and productive farming methods, (ii) access to credit for agro-dealers was facilitated, (iii) demand creation activities such as field days were supported and (iv) local agro-dealer associations were reinforced.

Various achievements have been reported from these and other interventions to support agro-dealers. First, the number of agro-dealers has increased in Kenya, leading to a large network of more than 10,000 agro-dealers [80]. This has, in-turn, reduced the distance travelled by farmers to secure farm inputs, and hence lowered search costs [81]. In addition, the initiatives reported to have supported agro-dealer capacity building in various areas such as business management and marketing; and supported in creating business linkages; creating agro-dealer associations; and facilitating improvement in access to finance [80,82].

### 3.2 Data collection

The data were collected in Kenya at the start of the 2019 principal maize growing season which ran from March to June. Eighty agro-dealers were interviewed across five maize growing agro-ecological zones (16 in each zone): highlands, moist transitional, wet lower mid-altitude, dry mid-altitude, and dry transitional. In each zone, two counties were selected randomly, leading to a total of ten counties. For each county, one sub-county was randomly selected and in that sub-county, four agro-dealers in the sub-county main center (referred to as urban center) and four agro-dealers in rural centers were randomly selected from a centralized agro-dealer database of Kenya. This database was verified and adjusted where necessary by local government officials at the sub-county level. The interview consisted of two parts: the agro-dealer survey and the independent store evaluation.

Additionally, intercept interviews were carried out with 466 farmers who purchased maize seed at an agro-dealer. Three counties were selected based on agro-ecological zone and prevalence of maize farming: Embu (dry mid-altitude), Kakamega (moist mid-altitude) and Trans-Nzoia (highlands). In each county, the county center and a rural location were selected and one to three agro-dealers were selected in each location to recruit farmers, depending on foot traffic. Locations of the intercept interviews were selected independent from the agro-dealer surveys.

Finally, semi-structured interviews were carried out at eight seed companies with company owners or managers in Kenya in 2019. These interviews lasted on average 2–3 hours and covered a range of topics including their seed portfolio, seed production and cost, varietal replacement, seed distribution and marketing. In this paper, insights on varietal replacement, seed distribution and marketing will be presented and discussed.

### 3.3 Agro-dealer survey

A structured questionnaire guided the agro-dealer interviews. These were carried out with the individual who was considered to be the most knowledgeable about maize seed sales by those working at the store on the day of the interview. This could have been the store owner as well as a store manager or an experienced employee. Interviews lasted roughly 60 min. The survey was extensively pretested before the maize seed-selling season and revised over several iterations. The questions included a mix of closed questions and more interactive questions. Flash cards were used to facilitate engagement with interviewees for multiple choice questions.

The survey covered five topics: (i) current maize seed varieties in store, (ii) selection of new maize seed varieties, (iii) promotion of new maize seed varieties, (iv) engagement with seed suppliers and customers, and (v) opportunities and challenges of the agro-dealer to increase maize seed sales. At the end of the survey we played a hypothetical investment game, to shed light on their priorities for investments if liquidity constraints were eliminated. They were offered 1 million Kenyan Shilling (about USD 10,000) to invest in various activities that would increase their maize seed sales according to the interviewee. The investment options were fixed, and they could invest 10 chips, each worth 1/10 of the total amount, in the different options to increase seed sales (Figure A1).

### 3.4 Store evaluation

Besides the survey, a second method was developed to collect observational data on the store environment for maize seed. During field visits for the design of this study, we recognized the considerable variation in the structure of agro-dealer shops and in how maize seed was sold to farmers. The marketing literature has long recognized the influence of store environment on quality perception and consumer purchase behavior [[83], [84], [85]]. For example, Spies et al. [85] found that a poorly designed store lay-out negatively affected customers’ experience. To quantify variation in store layout, an indicator was developed to objectively evaluate the different stores. Available literature has examined the highly professionalized Western retail sector, covering such indicators as music choice, ambient lightning and tasteful design [83], and thus provided limited support for considering how to evaluate the small, rural agro-dealer stores in Kenya that sold maize seed. Therefore, the authors used observational differences spotted during numerous agro-dealer visits across the country to develop the indicators.

Agro-dealer stores were evaluated on ten attributes, selected and tested by the authors. Table 1 lists these indicators as well as the evaluation criteria. The scoring ranged from one to five, where a score of one reflected ‘very bad’ and a score of five reflected ‘very good’. Scoring instructions were given for levels one, three and five where a rating of two or four would be given for scores judged to lie between these three levels. Scoring was done independently by the team leaders and each enumerator in the team. After each agro-dealer visit, scores were compared and where there was a difference in scoring within the team, consensus on the final score was reached through a discussion. The team trained extensively on this exercise, resulting in a high level of uniformity. In more than 85% of the cases, scores between the team leader and enumerator were similar. In most other cases, the scores were only one point different and consensus was easily reached.

**Table 1:** External evaluation factors and scoring description.

No	Factor	Scoring description		
		1 = Very bad	3 = Average	5 = Very good
1	**Price indication of maize seed***(how good price is indicated)*	No price indication	Some indication; not very visible	Very clear indication, clean and easy to spot
2	**Visibility of** maize seed (*how easy buyer can see* the available *varieties)*	Difficult to see	Visible but scattered in shop	All clearly visible; good arrangement
3	**Placement of maizeseedin the store***(Which place* does the seed *getin store?)*	Dark corner in the back; hard to spot	Not standing out; can be seen	On clear and prominent spot in store
4	**Proximity of the maize seed***(how close maizeseedis to buyer)*	Bales (contains 12 seed bags of 2kg) seen but no individual bags; no self service	Bags visible; not possible to touch; no self service	Bags within reach; can be touched; self-service
5	**Number of visible varieties***(how manymaize seed varietiescanbeseenfrom buyers point)*	1 or 2 varieties; from same company	Around 5 varieties.; some difference in companies	8 or more varieties; from at least 3 companies
6	**Targeted sales***(How big is effort to highlight one/some variety?)*	No variety standing out	Some effort to promote 1 variety over others	Strong effort to promote 1 variety over others
7	**Overall professionalism***(about store as a whole)*	Not clean; products on floor; debris; dark …	Effort to keep clean; some organization	Very clean; all organized; clear structure; staff uniforms
8	**Store visibility from outside***(clear it's an agro-dealer, that seed is sold; reference to maize)*	No or small sign; no clear indication that seed is sold	Clear signage, not professional; some info that seed is sold	Clear and professional signage; clear that seed is sold
9	**Seed marketing***(*how *a variety is promoted)*	No or old posters; no leaflets; no seed samples	Older posters; some leaflets; no seed samples	New posters in prominent place; pile of leaflets; seed samples
10	**Detail** variety info *(Is there any info about varietal performanceof maize seed?)*	No information	Some leaflets or info on posters; for a minimum of two varieties	Clear information on leaflets or posters; for all varieties in store

### 3.5 Intercept interview

Intercept interviews obtained direct farmer feedback on their purchase decisions. Respondents exiting the agro-dealer with at least one bag of maize seed were invited to participate in the 10–15-min survey. Given that interviewers were applied immediately after a farmer purchased seed, recall bias was minimized, if not eliminated altogether. The survey focused on seeds purchased, in store engagement on seed selection, and the underlying motivations for the purchase. Respondents received a small non-monetary incentive for their participation. Tents were erected directly outside of the agro-dealer to provide shade, as well as to reduce potential unease (and bias) among respondents when answering sensitive questions about seed and store selection.

Data was collected during 18 days in the field, with participant rates ranging from 5 to 49 per day per agro-dealer. Due to the tropical cyclone ‘Idai’ that redirected rains away from the subregion, the traditional starting period of the maize growing season was disturbed by scattered and absent rainfall resulting in longer seed purchase period. Due to lower than usual foot traffic, almost all farmers that came out of the shop when enumerators were present and met the screening criteria (i.e. being involved in varietal decision making as well as maize farming), participated in the survey. 53.4% of the participants were interviewed in urban locations, in 7 different agro-dealer stores, while 46.6% of the participants were interviewed in rural locations, in 13 different stores.

## 4 Results

### 4.1 Sample description

Table 2 presents descriptive information on the agro-dealerships (n = 80) and the farmers who participated in the intercept interviews (n = 466). Over 60% of the participants in the agro-dealer survey was male and the average age was 40. The vast majority of interviewees finished secondary education (90%), over half of them had attended college or university. Almost 70% of interviewees were owners and approximately 20% claimed to be a store manager. The agro-dealerships were, on average, eight years in business and most stores were owned by a sole proprietor. Approximately half of the stores reported the maize seed was one of the top three annual revenue generators and it was the most important annual revenue generator for roughly 10%.

**Table 2:** Characteristics of the agro-dealers and farmers participating in the study.

Agro-dealers		Farmers	
N	80	N	466
Gender (%)		Gender (%)	
Male	56.2	Male	66.3
Female	43.8	Female	33.7
Age in years	40.0 (12.3)	Age in years	48.3 (15.6)
Education level (%)		Education level (%)	
Higher than secondary	57.5	Higher than secondary	17.8
Secondary	32.5	Secondary	23.0
Lower than secondary	10.0	Lower than secondary	59.2
Position (%)		Farming experience in years	18.0 (14.6)
Owner	68.8	Size of maize field in acres	2.0 (2.7)
Manager	18.8	Part of maize harvest sold (%)	38.7
Employee	12.4		
Years in business	8.3 (6.7)		
Importance of maize in revenue (%)			
Most important	10.0		
In top 3	51.3		
Not in top 3	48.7		

Standard deviation in parantheses

The farmer sample of the intercept interviews consisted for two third of male farmers, whose average age was 48 years, and almost 60% of farmers had less than a secondary degree. Participating farmers had on average 2 acres allocated for maize farming and have been doing this for 18 years; less than half of the harvest from the main production season was sold as grain.

From the interviewed seed companies, five operated on a national level, two on regional level and one was a multinational. Maize seed sales contributed for over 70% of the revenue for five companies, of which two were only dependent on maize seed. The other companies had a more diversified portfolio including other grain or crop seed, pesticides, or fertilizer; or went into grain production. Maize seed production volumes ranged from 500 tons to 2500 tons, with one exception of 20,000 tons. The average number of varieties the companies were selling was five, ranging from two to seven varieties per company and one exceptional case that produced 15 different maize varieties.

### 4.2 Seed company engagement in retail and distribution

Distribution and sales strategies were strongly linked to the volume of seed produced by the seed companies. Smaller companies dealt directly with agro-dealers. For companies with larger volumes, this became too complicated and time intensive in terms of logistics and therefore preferred to use middlemen (large agro-dealers or distributors) to further distribute seed to local retailers. Local seed companies reported several challenges to build their retail and distribution networks. One issue mentioned by the Kenyan-owned companies was the strong competition represented by the multinational seed companies operating in Kenya. According to them, multinational companies invested larger budgets into seed marketing compared to their limited in scope and cash-constrained marketing operations. Another challenge mentioned was finding effective and reliable staff to lead the marketing and sales work. With maize seed being a seasonal product dependent on one or two peak moments per year, marketing and sales activities were concentrated in time, making it expensive to maintain a full marketing and sales division paid throughout the year focused on maize seed. With one exception, seed companies were owned by breeders and agronomists and their primary focus was on varietal development and seed production, and less on the business side of the company.

Launching a new hybrid onto the market was seen as a daunting undertaking by most seed companies. Reasons to update or expand the seed portfolio included better disease resistance of new material, higher expected yields for farmers but also lower production cost (linked to higher yields of parent seeds and synchrony in flowering of the male and female parent line). The priority of seed companies in terms of marketing and sales was on creating awareness about their products among farmers, mainly through demonstration plots or field days, radio adverts and free sample packs. Seed companies also felt they lacked institutional support with their distribution and promotion. One interviewee highlighted that they were responsible for all demand creation without support from extension services. Another major challenge with varietal turnover was the dilemma to reduce the availability of a successful hybrid in the market. One of the company managers mentioned that in the effort to replace a variety, farmers went to competing brands when they could not purchase the old hybrid, instead of switching to the new hybrid of their brand. Rotating out older varieties and bringing in new varieties was seen as a slow and careful process with considerable risks.

### 4.3 Supplier influence on agro-dealers

Table 3 reports the average buying price, selling price and margin of a 1 kg maize seed bag at the agro-dealer. The results showed that how maize seed was sourced by the agro-dealer, i.e. direct sourcing from the seed company or sourcing through a local distributor, had consequences for the agro-dealer as well as farmers who purchased the seed. Only one-fourth of the maize hybrids available for sale were directly sourced from seed companies, with most of the seed supply coming from local distributors. The average margin for the agro-dealer on a seed bag was 8.3% on a buying price of 2.06 $/kg.^1^ Not only were buying and selling prices significantly lower when the seed was sourced directly from a seed company, the agro-dealer margin was 23% higher compared to the margin they could have achieved if the seed had been sourced from wholesalers. In summary, the highest possible margin that could be achieved from the selling of maize seed (ie, when seed was sourced directly from seed companies) was low, and sourcing from middlemen led to even further reductions in agro-dealer profit.

**Table 3:** Average hybrid maize seed buying prices, selling prices and margins.

	Overall	Seed company	Local distributor	*t* value/Chi-square	*p*-value
Share of seed supply (%)		27.0	73.0		
Average buying price ($/kg)	2.06 (0.33)	1.86 (0.30)	2.12 (0.32)	−8.769	<0.001
Average selling price ($/kg)	2.23 (0.33)	2.07 (0.32)	2.27 (0.32)	−6.616	<0.001
Average margins ($/kg)	0.17 (0.11)	0.21 (0.11)	0.16 (0.11)	4.482	<0.001
Reliability of supply (%)				15.505	<0.001
Very reliable	70.5	82.1	66.2		
Averagely reliable	23.9	15.0	27.2		
Not reliable	5.6	2.9	6.6		

Standard deviation in parentheses.

Given that seed quality diminishes over time, agro-dealers need to carefully anticipate sales, or else run the risk of having leftover seed stock at the close of the seed selling season. Only 6.3% of our sample reported that their suppliers accepted leftover stock at the end of the seed selling season, with the overwhelming majority reporting that leftover seed remained with them. The most frequently mentioned tactic to avoid having on hand unsold stock at the end of season was restocking in small amounts as per demand (Table 4). As discussed further, this might also have been driven by liquidity constraints and not solely due to the fear of leftover stock. Few reported the use of marking tactics, such as selling at discounts at end of season or intensive promoting of seed at end of season—not altogether surprising given the small margins earned from maize seed sales and the lack of direct engagement with seed companies.

**Table 4:** Agro-dealer efforts to reduce leftover stock of maize seed at the end of the season.

Restock maize seeds in small amounts as per demand	75.0%
Buy only enough maize seeds for the season	56.3%
Sell maize seeds at discount near end of the season	6.3%
Intensive promotion of remaining maize seeds towards end of the season	6.3%

### 4.4 Agro-dealer challenges and priorities

**Fig. 3:**
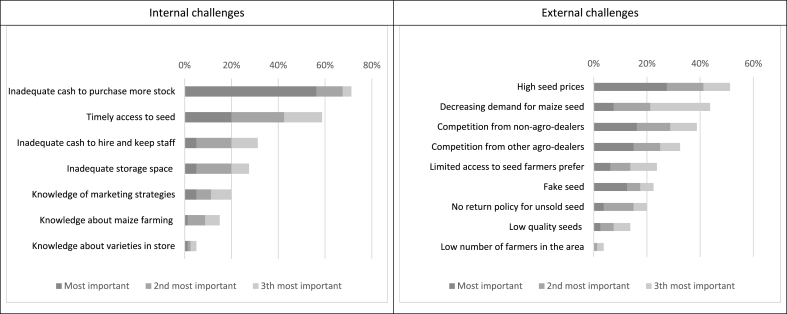
Agro-dealers’ perceptions of their challenges to sell hybrid maize seed (n = 80)*. *Agro-dealers could choose their top 3 most *important challenges from a predefined list of options.*

Fig. 3 reports the most important internal and external perceived challenges by the agro-dealers to increase their maize seed sales. Among the internal challenges identified, liquidity constraints to increase seed stock were the most prevalent. Secure and timely access to maize seed was the second key constraint identified. The third identified internal challenge was to hire and maintain qualified sales staff. Limited knowledge of the agro-dealer about maize seeds sold in store, farming practices, or marketing strategies was not perceived to be a barrier to increase maize seed sales by most agro-dealers.

The main perceived external challenges to increase maize seed sales were related to the cost of seed and the ratio of seed buyers versus suppliers. Agro-dealers faced strong competition from official retailers as well as unofficial seed distributors (for example seed being sold on the market), and over 40% referred to less foot traffic in store as a major external challenge. Seed quality did not seem like a major barrier, both fake seed and low-quality seeds were only mentioned by a small fraction of the sample.

To gain insights into potential investment priorities, the agro-dealers survey explored the hypothetical question: what investments would the agro-dealers make to increase seed sales if financial constraints were lifted (i.e. agro-dealers received a hypothetical grant). Table 5 reports the results of the investment exercise. The most preferred method to increase seed sales was to increase stock of the currently available maize varieties, mentioned by almost every agro-dealer with an average investment of one-third of the resources available. Besides increasing current stock, other popular efforts were focused on improvement of the store itself, i.e. investing in storage space and improving the lay-out of the store. Ten percent or less of the hypothetically available resources would have been used to increase product portfolio with new maize hybrids and to promotion methods such as workshops, demo-plots or radio advertising.

**Table 5:** Agro-dealer investment preferences for increasing maize hybrid seed sales.

	% of agro-dealers investing in this	Share of investment
Increase stock of currently available maize varieties	93.7%	33.6%
Invest in storage space	68.7%	13.0%
Improve looks of the store (new shelves, better signage)	67.7%	11.3%
Add newly released maize varieties to stock	62.5%	10.0%
Organize farmer workshops to promote seeds	58.7%	9.3%
Invest in bigger/more demo plots	55.0%	8.0%
Invest in radio/TV advertising	50.0%	6.6%
Hire additional staff	40.0%	4.5%
Provide credit to farmers for more seed purchases	15.0%	2.0%

### 4.5 Store environment for maize seed sales

**Fig. 4:**
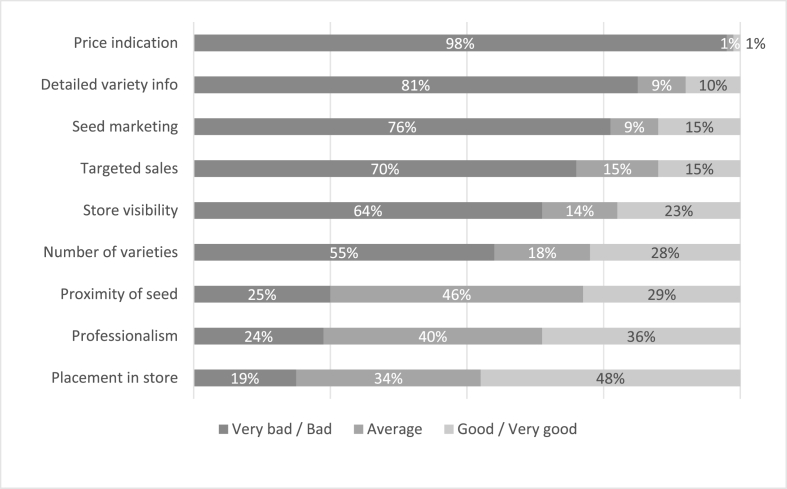
Agro-dealer retail space evaluation on selected attributes (n = 80).

Fig. 4 shows the results of the store evaluation. Looking across all the indicators, roughly 18% of the agro-dealers scored on average below 2 (between very bad and bad), 63.8% had an average score between 2 and 3 (between bad and average) and 18.7% above 3 (between average and good). The factors with the lowest scores in the store evaluation were price indication and the availability of any marketing or technical information of the stocked hybrids. Except for two agro-dealers, none provided information on prices for the different maize seed products sold in the store. Four out of five agro-dealers had practically no visible information in the form of posters or leaflets about the available seed offer and less than one out of three agro-dealers aimed to promote one particular variety over the others. The agro-dealers scored well on factors associated with ensuring that seed was positioned in a prominent place in the store, variety labels were clearly visible, and the packs were accessible for customers.

### 4.6 Engagement between agro-dealers and farmers

**Fig. 5:**
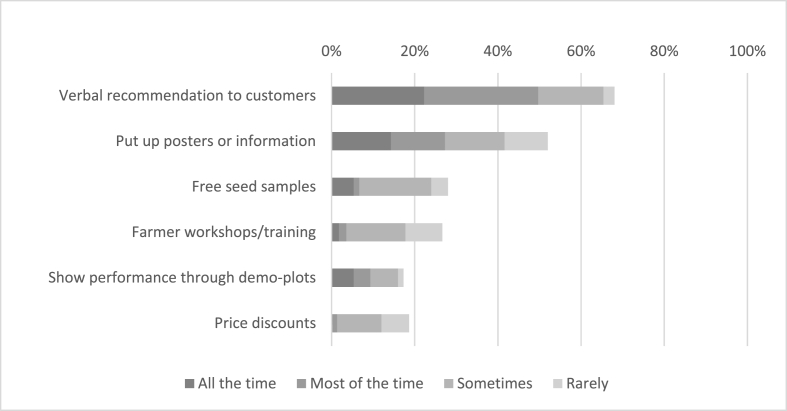
Agro-dealer selected actions to promote new hybrids and frequency of actions asked on a 4-point scale ranging from all the time to rarely (n = 80).

Fig. 5 shows the actions of agro-dealers to promote a new variety in their store. Interviewees that engaged in seed promotion tended to limit their efforts to verbal recommendation of new products; around one quarter of the sample regularly hung posters of new hybrids in the store. More engaging efforts to promote new varieties such as free seed samples, workshops, demo-plots or price discounts were barely used by agro-dealers.

Table 6 reports farmer attitude and behavior towards his/her maize seed purchase including preparation before seed purchase, attitude towards seed selection and behavior in store for seed purchase. Approximately half of our sample reported that they did not discuss seed selection in the household, 10% reported a thorough discussion on seed selection and 40% a light discussion. Subjective knowledge about the maize seed offer was limited, with only one out of five of farmers considering themselves to have had sufficient (14.6%) to very good knowledge (5.6%) about the available seed offer. Thirty-five percent considered themselves to have had sufficient knowledge about some of the varieties and the knowledge on seed of almost 45% of the farmers was restricted to what they grow.

**Table 6:** Farmer attitude and behavior towards seed selection at the agro-dealer (n = 466).

Preparation before seed purchase			

Subjective knowledge about varieties in store (%)		Discussion at home about what seed to buy (%)	
I only have knowledge about the maize I purchased today	18.5	No discussion	48.1
I have some knowledge about some other varieties than the one I bought	26.4	Short discussion	40.1
I have sufficient knowledge about some of the varieties in the store	35.0	Detailed discussion	11.8
I have sufficient knowledge about most of the varieties in the store	14.6		
I have a very strong knowledge about all varieties in the store	5.6		

Attitude towards varietal selection			

Degree of certainty about which variety to purchase before entering the store (%)		Interest in other varieties (%)	
100% sure	82.6	I have no interest in other varieties	49.6
Very sure	4.9	I have very little interest in other varieties	28.1
Fairly sure	9.0	I am somewhat interested in other varieties	16.5
I was not that sure	1.7	I am very interested in other varieties	5.8
I had no idea	1.7		

Behavior in store			

Attention to varieties in store (%)		Information requested from agro-dealer about maize seed (%)	
I did not look at the other varieties than the one(s) I bought	75.1	Yes	12.7
I had a quick look, but I am not able to tell what all the other varieties are	15.0	No	87.3
I looked at the available offer	8.4		
I had a detailed look at each variety	1.5		

Farmers showed a rather passive attitude towards seed selection. Most farmers entered the store with a fixed idea of what seed they would buy and expressed very little interest in changing their idea. This conservative attitude was also reflected through in-store behavior of farmers. Almost nine out of ten farmers did not ask any questions about the other varieties then the variety they purchased. Also engagement with the surroundings in the store offer was minimal. Three quarter of our sample did not look at the seed offer available and another 15% said that they only had a quick look but wouldn't be able to tell what the other varieties are. Only 10% of farmers reported that they examined the available maize seed offer while in the store.

## 5 Discussion

### 5.1 Challenges for value chain development

This study aimed to better understand the challenges for maize seed value chain development. We started by exploring three questions related to the needs, realities and interactions of farmers, agro-dealers, and seed companies: i) how farmers decide on their seed purchases and engage with retailers, ii) what challenges agro-dealers face to support innovation and value adding and iii) what the incentives, capacities and limitations of seed companies are to build or expand their market. We address each of these questions in turn, while also exploring the implications of the answers in terms of possible entry points for supporting maize seed value chains in Eastern Africa.

#### 5.1.1 Farmers (seed customers)

An understanding of how farmers decide on seed purchases and how they engage with retailers is key to better assessing the options available for advancing new hybrid seed varieties. Evidence here suggested that farmers were not seeking out new technologies: most of them came to purchase the variety they knew and trusted, without looking at the available stock. A common argument in research on maize seed adoption has been that low hybrid uptake and the persistence of older hybrids in the market reflected farmers’ lack of information about the quality and traits contained in hybrids--issues which could be resolved, in part, by better informing farmers about hybrids [48,49,51].

The findings of this study suggest that additional elements are at play—elements related to loss aversion and reference dependence. A large body of literature in consumer psychology theorizes that individuals use two cognitive processes when making decisions: ‘system-one thinking’, characterized by fast, instinctive and emotional decisions, versus ‘system-two thinking’, which is considered to be slower, more deliberative and logical [[86], [87], [88]]. Everyday decision making is generally dominated by system-one thinking, while system-two thinking only takes over when default decision making feels wrong [89]. If seed purchasers were to apply a system-two decision process for seed purchases, then the lack of knowledge about the available seed options would reinforce the unengaged decision making [90,91], and more information on seed attributes potentially favors the purchase of new hybrids. However, our results suggest the possibility that farmer attitudes and behavior regarding seed decision might be more in-line with system-one type thinking. Unless farmers experience first-hand disappointment with varietal performance (for example low germination) or withdrawal of their preferred variety from the local market, farmers have little incentive to question their seed choice and assume the risks related to poor performance of a new seed. This implies that nudging farmers towards new seeds might require much more implicit persuasion methods, meaning multi-dimensional strategies for and extensive investment in seed marketing. In some countries, multinational seed companies have long recognized the system-one nature of maize seed purchase decisions, investing heavily in building brand recognition and farmer trust, and, in some cases, charging substantial price premiums as a result.

The findings regarding farmers also question the logic behind the prevailing theory of change for maize seed systems development, i.e. the assumption that better seed will automatically lead to changes in farmers' seed purchase behavior. For example, Glover et al. [92] question if the highly anticipated Golden Rice variety, a genetically engineered rice variety developed to address Vitamin A deficiency, still has a chance on the market as the transgenic trait has been transferred in two outdated rice varieties that are no longer competitive. Within many public breeding programs supported by CGIAR, efforts to incorporate a stronger end user focus has been made more explicit through the introduction of ‘product profiles’ [93,94]. Product profiles are defined as a trait package a new variety is expected to have to serve a specific market segment (meaning a group of users who are considered to have a relatively homogenous demand for a product or service). This paradigm shift in breeding strategies towards end-users is a necessary for step value chain development and Britwum et al. [95] provide evidence that replacing a popular variety can only be done if a new product mimics key attributes of competing varieties, in addition to better performance on other attributes. However, our results showed that having the right product alone may not be sufficient to change farmer purchase decisions. More effort is needed to overcome the influence of loss aversion and reference dependence, among other factors, that influence farmers' decision process for hybrids.

#### 5.1.2 Agro-dealers (retail)

This brings us to the incentives, capacities and limitations of retailers and their engagement downstream (i.e. with farmers who purchase seed) and upstream (i.e. with seed businesses and seed wholesalers). Our findings casted doubt regarding the willingness of retailers to deliberately and actively promote new hybrids. Besides engagement with farmers who requested advice on seed selection, retailers made little, if any, effort to actively promote maize seed in their store during the maize seed selling season. This finding echoes with that of a recent agro-dealer survey which found that the total budget invested in promotion accounted for less than 1% of total expenses [96]. Our retail scan showed how unconducive the retail space was for farmers to select maize seed. In most cases, the retail space lacked dedicated spaces for maize seed sales, exhibited poor lighting where seed was sold, and provided no promotional material on seed options nor clear indications of seed prices. The latter suggesting a serious limitation for promoting seed based on price, a common practice applied in the marketing of new products. The overall agro-dealer approach to seed sales responds to the system-one decision making process exhibited by farmers: given that farmers purchase seed in an instinctive manner, only the most rudimentary effort is made to actually promote maize seed.

Moreover, agro-dealers lacked the financial resources to expand their sales of seed and other products and faced bleak options for building their asset base over time. Liquidity constraints, combined with low margins from seed sales and strong competition among retailers, implied that few agro-dealers would have been able or willing to invest in in-store marketing. The outcomes of the hypothetical investment game also showed that agro-dealers had little interest to invest in seed marketing if additional funds were available. Given the small volumes of seed typically handled by an agro-dealer, combined with limited cash flows, few agro-dealers were willing or able to make bulk seed purchases directly from seed companies. By sourcing seed from middlemen agro-dealers reduced their already low margins from seed sales, an issue that was also raised by Goedde et al. [97]. Besides low margins on seed sales, agro-dealers faced considerable financial risk in maize seed sales. Maize seed is not only seasonal, but also diminishes in quality as prolonged storage at ambient temperature reduces germination rates [98]. Within the short selling window, the agro-dealers needed to have enough stock to deal with peak demand, while at the same time avoid being left with an unsold perishable product at the end of the buying season. The most popular strategy of agro-dealers to avoid leftover stock, also driven by cash constraints, was to restock weekly in small amounts.

#### 5.1.3 Seed companies (production)

Results suggest that addressing the varietal turn over issue in maize seed value chains will require more active engagement by seed companies on two fronts: direct efforts to increase awareness and demand for new hybrids as well as stronger engagement with agro-dealers to encourage growth of new varieties. Rutsaert and Donovan [59] described how seed companies in Kenya focused on increasing awareness and triggering farmer demand through demonstration plots, radio advertising and seed trial packages. However, seed companies tended to pay little attention to their retail network during most of the year. The seasonal nature of seed sales results in marketing and sales becoming a seasonal activity that requires considerable financial and human resources over a relatively short period of time. Where these resources are not available, seed companies rely heavily on middlemen for seed distribution and agro-dealers for in-store promotion and sales. In cases where a variety is established and sought out by farmers, this approach can be efficient and effective. However, where this is not the case the path to achieving recognition in the retail market becomes less certain, as retailers would be needed to actively promote new varieties, despite their limited incentives to do so.

This brings us to another important issue for development of the maize seed value chain: the role of price in signaling quality and unique attributes in seed. From a development perspective, greater farmer access to a diversity of affordable maize seed represents a desirable outcome from public investments in breeding. From a value chain perspective, however, the goal of seed businesses and the incentive for retailers is to transform the discussion from affordability to value adding (and related capacity to capture higher prices and profits). Following that principle, a variety with better qualities and higher potential returns should have a higher intrinsic value and higher prices could be instrumental in communicating the increased value. However, recent evidence shows that smaller-scale, locally owned seed companies tended to set seed prices lower than those of well-known international seed companies [33]. This raises questions about the incentives and capacities of smaller seed companies to update their seed portfolio if related investments cannot be translated into higher returns. If seed prices are constraint, the only way to increase margins for a company is to reduce production costs, which was an important reason for seed companies to invest in a new variety.

### 5.2 Research and development shift towards seed value chains

Looking forward, research on seed systems development and varietal turnover should do more efforts to take into a value chain perspective on the seed sector, acknowledging the actors involved and how seed is reaching the farmers. Since the early 2000s, research has explored varietal and trait preferences of farmers through participatory varietal selection methods [22,99,100] and revealed preference methods (e.g. choice experiments and experimental auctions) [[101], [102], [103]]. These studies aimed to predict the demand for new varieties or traits by confronting respondents with a number of trade-offs and expecting them to make a rational decision on the ‘best’ seed for their needs. Our results, however, suggest that farmer decision making in store is more intuitive than deliberate. While preference studies can provide useful insights on farmer needs, we question if they are useful predictors of farmer demand. Our study brings to light the urgent need for solutions to the marketing challenges for maize seed. This implies experimental research designs in-store, which can result in more realistic insights into the seed purchasing decision. Hoffmann et al. [104] presented an interesting example, whereby marketing campaigns for aflatoxin labeling on maize flour in Kenya were tested in store and the impact was measured through sales data. Secondly, research should explore how to influence intuitive farmer decision making by overcoming loss aversion and reference dependence of farmers. Heiman and Hildebrandt [105] provide an overview of marketing techniques in agriculture that can play a role in reducing pre-purchase risks. Currently, the most frequently used method in seed systems are demonstration plots which aim to reduce uncertainty about biological and agronomic innovations; but have little influence on buying behavior [106].

From a development perspective, there is also a need to better understand the drivers and challenges for innovation within the seed industry. The Access to Seed Index [107] as well as The African Seed Access Index (TASAI) are two initiatives that started in the last decade to gain a better understanding of overall seed sector development and both have contributed to better macro-level understanding of the seed sector landscape in the Global South. We argue that there is also a need to complement these initiatives with indicators that track micro-level changes in seed markets, captured through case studies of efforts to introduce new hybrids in the market. How do companies manage their product portfolio and balance investments in new varieties; what are the best ways to expand sales to a new area; how to build up efficient distribution networks in an extensive and fragmented retail landscape, what growth can be expected for new varieties and how does this growth correlate with investments in marketing and promotion? To our knowledge, this information has not been captured in ongoing efforts and could provide very useful insights to the discussion on varietal turnover.

Third, there is a need to create an enabling environment for value chain development and varietal turnover. An assessment should be made of what can be expected of the key actors (i.e. the seed producers and distributors) and where external support is required. Results showed that local seed companies as well as agro-dealers lacked the capacity to invest in marketing and sales of new products. Along the lines of early generation seed support, one could explore the potential of a third party that acts as a service provider to the sector and supports companies with their marketing and sales strategy as well as the rollout. Another challenge is the lack of marketing intelligence and demand estimations, which limits the capacity of companies to develop plans for future growth. Multinationals are often better equipped to invest in this but for smaller companies, there could be a role for the development sector to provide intelligence on seed demand and market competition.

## 6 Conclusion

The limited success of modern crop breeding programs and recent innovations in the seed sector in general warrants a different approach going forward than only focusing on supply of new hybrids. Given the complexities of seed marketing and the realities of chain actors, the development process will require careful planning and strong engagement on the ground. Due to its strong hybrid vigor and benefits for farmers to purchase annually, maize has been one of the crops with high private sector interest. Although it has been seen as an example crop for formal seed sector development, uptake of new stress tolerant varieties has not been in line with expectations. Looking specifically at the issue of slow varietal turnover, advancement warrants new forms of inter-business relations, combined with innovation in marketing and information systems. There is a need for broader (multidisciplinary), more reflective, programming, which to date, has yet to emerge in the context of formal maize seed systems. However, these issues are not limited to maize seed alone with other crops progressing towards increased private sector involvement. Demand orientation needs to go beyond assessing farmer preferences or targeted breeding programs. Building seed value chains will require much more attention in development research, focused on understanding needs of the stakeholders involved as well as strengthening their capacity to drive innovation.

## Author statement

Pieter Rutsaert: Conceptualization, Data collection, Data Formal analysis, Writing – original draft, Writing – review & editing, Jason Donovan: Conceptualization, Data collection, Writing – original draft, Writing – review & editing, Simon Kimenju: Data collection, Data Formal analysis, Writing – original draft

## Footnotes

1 Exchange rate on 18 March 2019 (start of the field work): 1 USD = 99.9 KES
