# Supplementary figures and images for: Demand-side challenges to increase sales of new maize hybrids in Kenya
Source: Technol Soc. 2021 Aug;66:101630. doi: 10.1016/j.techsoc.2021.101630 (PMC8374426; doi:10.1016/j.techsoc.2021.101630)

**Appendix**

Figure 1A : Material used for the agro-dealer investment game


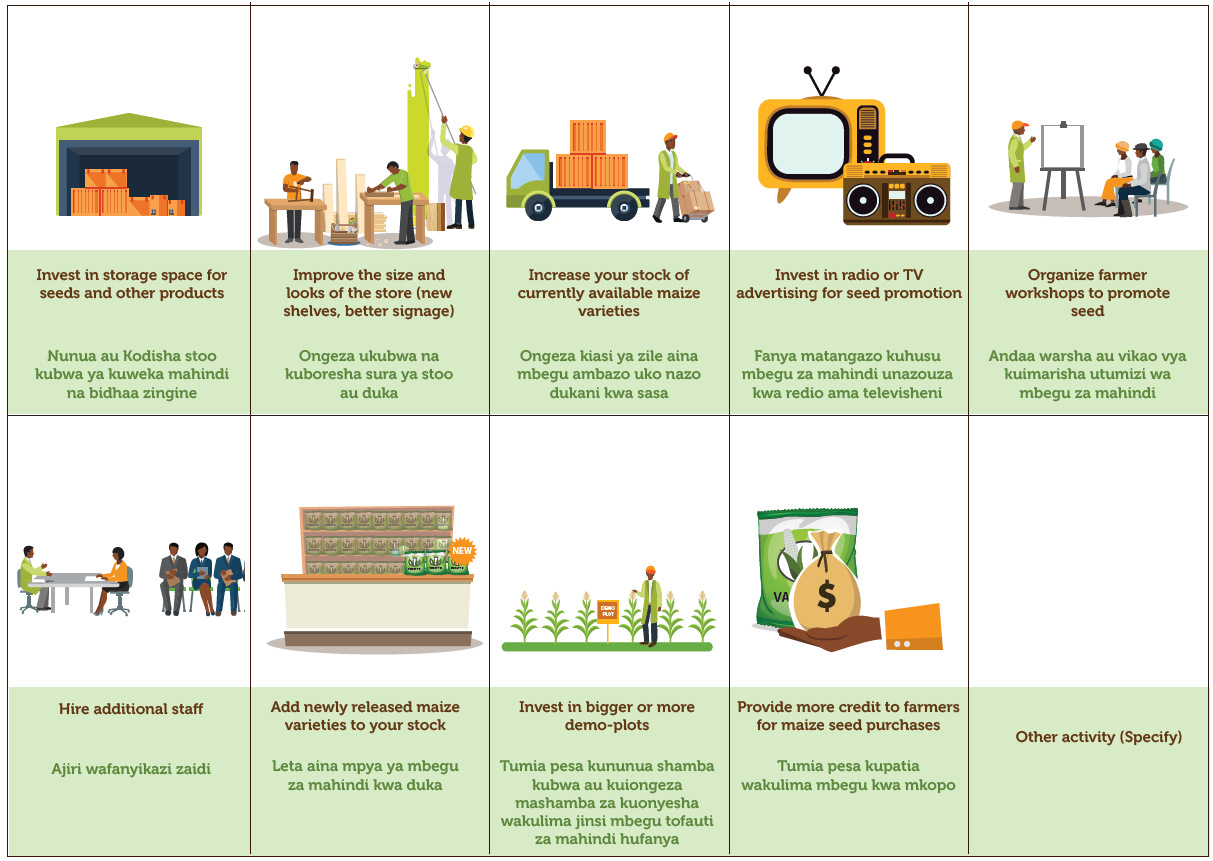

Supplement: Multimedia component 1 [file mmc1.docx]
